# The Beginning of the End: Initial Steps in the Degradation of Plasma Membrane Proteins

**DOI:** 10.3389/fpls.2020.00680

**Published:** 2020-05-21

**Authors:** Maximilian Schwihla, Barbara Korbei

**Affiliations:** Department of Applied Genetics and Cell Biology, Institute of Molecular Plant Biology, University of Natural Resources and Life Sciences, Vienna, Vienna, Austria

**Keywords:** ubiquitin, ESCRT (endosomal sorting complex required for transport), CME, plasma membrane protein, degradation

## Abstract

The plasma membrane (PM), as border between the inside and the outside of a cell, is densely packed with proteins involved in the sensing and transmission of internal and external stimuli, as well as transport processes and is therefore vital for plant development as well as quick and accurate responses to the environment. It is consequently not surprising that several regulatory pathways participate in the tight regulation of the spatiotemporal control of PM proteins. Ubiquitination of PM proteins plays a key role in directing their entry into the endo-lysosomal system, serving as a signal for triggering endocytosis and further sorting for degradation. Nevertheless, a uniting picture of the different roles of the respective types of ubiquitination in the consecutive steps of down-regulation of membrane proteins is still missing. The *trans*-Golgi network (TGN), which acts as an early endosome (EE) in plants receives the endocytosed cargo, and here the decision is made to either recycled back to the PM or further delivered to the vacuole for degradation. A multi-complex machinery, the endosomal sorting complex required for transport (ESCRT), concentrates ubiquitinated proteins and ushers them into the intraluminal vesicles of multi-vesicular bodies (MVBs). Several ESCRTs have ubiquitin binding subunits, which anchor and guide the cargos through the endocytic degradation route. Basic enzymes and the mode of action in the early degradation steps of PM proteins are conserved in eukaryotes, yet many plant unique components exist, which are often essential in this pathway. Thus, deciphering the initial steps in the degradation of ubiquitinated PM proteins, which is the major focus of this review, will greatly contribute to the larger question of how plants mange to fine-tune their responses to their environment.

## Endosomal System of Plants

**FIGURE 1:**
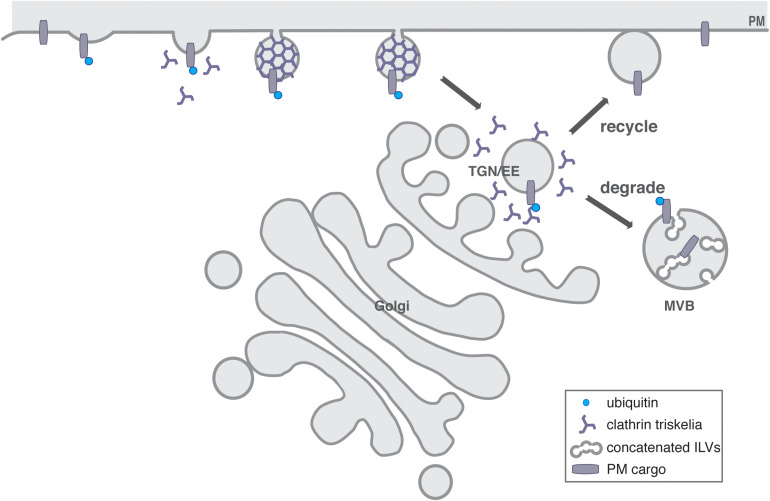
General overview of the endosomal system of plants. CME drives endocytosed cargo delivery from the PM to the TGN, which acts as an EE in plants. Here, the decision is made to either recycle the PM proteins and transport them back to the PM, or to send them for their degradation in the vacuole via the concatenated ILVs of MVBs.

In eukaryotes, next to transcriptional control, the regulation of protein abundance and localization at membranes is achieved by a complex system of internal membranes, the endomembrane system. This transports proteins to their site of action and provides spatial organization by compartmentalizing distinct cellular activities. Organelles of the endomembrane system are linked by branched and often bidirectional trafficking routes. The endomembrane system consists of two complimentary trafficking pathways, where the endocytic pathway is a major set of trafficking routes with sorting, recycling, and degradative functions while the secretory pathway exports proteins from the ER via the Golgi apparatus to the *trans*-Golgi network (TGN), where they are sorted for delivery to the plasma membrane (PM), to endosomes, or directly to lysosomes/vacuoles (see Figure 1; reviewed in Bassham et al., 2008; Frigerio and Hawes, 2008; Robinson et al., 2008; Richter et al., 2009; Viotti et al., 2010; Korbei and Luschnig, 2013; Paez Valencia et al., 2016).

In mammalian cells, the endocytic pathway includes early, recycling and late endosomes, while in yeast and plants, the TGN also serves as early endosome (EE) and recycling endosome, reflecting the ancestral organization of the endomembrane system (Dettmer et al., 2006; Lam et al., 2007; Day et al., 2018). Thus, the TGN/EE is a major sorting hub, which exists close to the *trans*-Golgi face, but also as a Golgi-independent compartment (Viotti et al., 2010; Kang et al., 2011). Here the two trafficking routes meet, sorting and recycling material to or from the Golgi, the PM and the lytic pathway (Figure 1) (Viotti et al., 2010).

## Endocytosis

Through an essential process termed endocytosis, cells internalize regions of the PM and apoplast into small endocytic vesicles, to remove PM-localized proteins, lipids, and nutrients from the cell surface (Henne et al., 2010). This mechanism is vital not only for basic cellular functions but also for growth, development and to be able to respond quickly and accurately to stimuli. During constitutive endocytosis, components are removed from the cell surface in the absence of any stimulus, while during ligand-induced endocytosis, specific proteins are internalized from the PM in response to a stimulus (Traub, 2009; Ekanayake et al., 2019). Endocytosis can be either dependent on clathrin or independent of it (Robinson, 2015; Sandvig et al., 2018). The coat protein clathrin, was first identified as being the major protein making up the lattice-like coat around vesicles in pig brains (Pearse, 1976) and in plants in soyabeans (Mersey et al., 1985). It builds a coat around the endocytosed vesicle to form a clathrin coated vesicle (CCV), which can form both at the PM and the TGN/EE in plants (Robinson and Pimpl, 2013; Rosquete et al., 2018).

Clathrin self-assembles into a three-legged triskelia composed of three clathrin heavy chains (CHCs) and three clathrin light chains (CLCs), that interact with the heavy chains (Chen et al., 2011; Robinson, 2015). The *Arabidopsis* genome contains two *CHC* genes, namely *CHC1* and *CHC2*, which are ∼90% identical and are functionally partially redundant (Kitakura et al., 2011). Their respective single mutants, however, show different phenotypes, where *chc2* plants display abnormal embryo and seedling development, but *chc1* mutants show no obvious phenotypes (Kitakura et al., 2011). The overexpression of a dominant-negative *C*-terminal hub of CHC competes with the unimpaired CHC for CLC binding and thus interferes with endocytosis of several integral membrane proteins, as well as the uptake of the endocytic tracer FM4-64 (Kitakura et al., 2011), highlighting clathrin-mediated endocytosis (CME) as predominant route for endocytosis in plant. Furthermore, a novel chemical inhibitor was shown to disrupt endocytosis by binding to CHC, which then fails to be assembled into coat structures (Dejonghe et al., 2019). The three *CLC* genes (*CLC1* to *CLC3*) show a partial functional redundancy, where the *clc1* plants show pollen lethality, but *clc2* and *clc3* single mutants are viable but with shorter roots and longer hypocotyls. The *clc2/clc3* double mutant is impaired in CME and displays strong developmental defects especially in auxin signaling (Wang et al., 2013; Yu Q. et al., 2016). GFP-tagged CLC2 localizes to the TGN, endosomes, and dynamic foci at the PM (Konopka et al., 2008; Ito et al., 2012).

### Clathrin-Mediated Endocytosis

Clathrin-mediated endocytosis is the most prominent endocytic pathway in plants and animals (McMahon and Boucrot, 2011) but was demonstrated only fairly recently in plants (Dhonukshe et al., 2007). It thus also represents a major mechanism for regulating signaling, plant immunity and the global responses, as many important PM proteins are established cargos for the CME pathway (Dhonukshe et al., 2007; Barberon et al., 2011; Di Rubbo et al., 2013; Mbengue et al., 2016; Yoshinari et al., 2016). Nevertheless, detailed descriptions of the events that make up CME in plants are still often based on the more advanced studies of CME in animal and yeast systems (reviewed in McMahon and Boucrot, 2011), although pronounced differences exist. Actin in plant CME for example is not present or required during CCV formation on the PM, but is critical for the early post-endocytic trafficking (Narasimhan et al., 2020).

### Five Steps of Clathrin-Mediated Endocytosis

**FIGURE 2:**
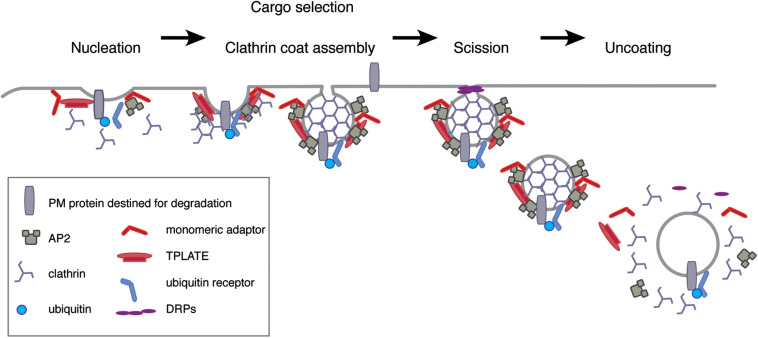
Five-steps of CME. At the PM, extracellular material is internalized by multiple means, including CME. Initiation of CME starts with the enrichment of cargo at nucleation site foci called CCP, which employs various adaptor complexes like TPLATE and AP-2 complexes but also monomeric adaptors. The selection of cargo might require association with thus far uncharacterized cargo-specific adaptor proteins. Adaptor-association is a prerequisite for the subsequent events of clathrin recruitment and coat assembly during the later stages of CCV formation. Mature CCVs are then cleaved from the PM (“scission”) by means of DRPs, thereby initiating the endocytic sorting of cargo-loaded CCVs. Before being able to fuse with the TGN/EE, the resulting CCVs undergo uncoating.

Clathrin-mediated endocytosis is a complex process that can be divided into five steps: nucleation, the packaging of cargo into the vesicle, clathrin coat assembly, the release of the mature vesicle from the PM or membrane scission, and uncoating including the fusing of the vesicle with endosomes (Figure 2) (McMahon and Boucrot, 2011). It starts with nucleation site foci at the PM called clathrin coated pits (CCPs), which eventually mature and bud off to form CCVs. These are uncoated and fuse with the TGN/EE, where the cargo is further sorted, either for recycling or degradation. The biogenesis of plant CCVs requires the functions of the clathrin core components, adaptors, linking clathrin to cargos and the PM, as well as accessory components, required for scission and uncoating events (Figure 2) (Chen et al., 2011; Korbei and Luschnig, 2013; Paez Valencia et al., 2016; Reynolds et al., 2018; Ekanayake et al., 2019).

#### Nucleation

Nucleation, or the budding of a vesicle, starts by the bending of the PM toward the cytoplasm and the formation of a membrane invagination called CCP (Figure 2). As clathrin does not interact directly with membranes or cargos, the formation of the CCP begins with the recruitment of adaptors proteins. These, interact with specific lipids on membranes as well as sorting motifs on cargo proteins and then bring clathrin to the initiation site at the PM (Figure 2). Thus, the starting point of CME depends on lipids, cargos, and adaptor proteins, which provides specificity, determining which cargo is selected and the subcellular compartment for subsequent trafficking. Adaptors therefore consist of multiple domains and motifs that provide a platform for interactions. In monomeric adaptors, these functions are contained within the same protein, while multiple subunits need to come together in multimeric adaptors (McMahon and Boucrot, 2011; Reider and Wendland, 2011; Cocucci et al., 2012; Paez Valencia et al., 2016; Reynolds et al., 2018).

In mammalian cells, nucleation is started by the binding of a complex composed of the adaptor protein-2 (AP-2) complex and 1–2 clathrin triskelia. This already preassembled complex is recruited to the PM by the membrane lipid phosphatidylinositol-4,5-bisphosphate [PtdIns(4,5)P_2_] (Cocucci et al., 2012). Following AP-2 and clathrin recruitment, monomeric adaptor proteins associate with and stabilize the curvature of the budding vesicle and clathrin coat (McMahon and Boucrot, 2011; Cocucci et al., 2012). These including the E/ANTH (epsin/AP180 *N*-terminal homology) domain proteins epsin and AP180, the EGFR pathway substrate 15 (Eps15) proteins as well as muniscin proteins like the FCH domain only (FCHo) proteins and intersectins (Cocucci et al., 2012). The E/ANTH domain is a conserved module for introducing curvature to the bound membranes, which binds phospholipids and proteins responsible for targeting of these proteins to specific compartments (Legendre-Guillemin et al., 2004). Initiation stages may involve the formation of a putative nucleation modules, which include FCHo, Eps15, and intersectins (Henne et al., 2010; Hollopeter et al., 2014) and define the sites on the PM where vesicles will bud (Mayers et al., 2013). However, adaptors might not be crucial for membrane bending in all CME events, as protein crowding at PM foci may be enough to bend the membranes (Dannhauser and Ungewickell, 2012).

#### Packaging of the Cargo

Regulation of PM protein abundance is based on the ability of the CME machinery, specifically the adaptors, to recognize proteins to be internalized through their specific motifs in their cytosolic domains and package them into the endocytic vesicles. There can be more than one sorting signal in the same cargo protein, participating in cargo recognition. These signals can be present in the protein sequence or structure, or can occur through covalent posttranslational modifications (Traub, 2009).

#### Clathrin Coat Assembly

The clathrin coat is assembled as soon as cargo is selected and bound by the AP-2, the plant-specific TPLATE complex, or by other cargo-specific adaptor proteins (Chen et al., 2011; Zhang et al., 2015; Reynolds et al., 2018). Clathrin triskelia are recruited directly from the cytosol, where they assemble into a regularly shaped lattice around the forming vesicle (McMahon and Boucrot, 2011). In mammals, polymerization of clathrin results in stabilization of curvature and displacement of some of the monomeric adaptor proteins and curvature effectors, such as Eps15 and epsin, to the edge of the forming vesicle (Tebar et al., 1996). Whether a similar mechanism exists in plants still needs to be determined.

#### Membrane Scission and Vesicle Release

The molecular scission that mechanically constricts the neck between the CCV and the PM is mediated by dynamins and dynamin-related proteins (DRPs). DRPs are large GTPases, that control membrane scission and tubulation (McMahon and Boucrot, 2011). Plants have six types of DRP families, of which the plant-specific DRP1 family and the DRP2 family, which is the most similar to metazoan dynamins, are involved in clathrin-mediated trafficking and CME (Bednarek and Backues, 2010; Fujimoto and Tsutsumi, 2014). A dominant-negative variant the dynamin related protein DRP1A inhibited endocytosis of the boron efflux transporter BORON TRANSPORTER 1 (BOR1) and altered its polar localization and vacuolar trafficking (Yoshinari et al., 2016).

In animals, Src homology-3 (SH3) domain containing proteins bind dynamin and recruit them to CCPs (Ringstad et al., 1997). In plants the TPLATE complex contains two subunits with SH3 domains, which have been shown to interact with members of the DRP2 and DRP1 families (Gadeyne et al., 2014). Furthermore, the SH3 domain containing protein-2 (SH3P2), which contains a BAR domain that induces vesicle tubulation, forms a complex with a DRP1, affecting its accumulation at the cell plate (Ahn et al., 2017). Whether such an interaction between SH3P2 and DRP1 could also have a significance for CME remains to be resolved.

#### Vesicle Uncoating

Shedding the coat of the vesicles allows components of the CME machinery to be recycled for further rounds of endocytosis and frees the vesicles for fusion with TGN/EE (Chen et al., 2011; Zhang et al., 2015; Reynolds et al., 2018). While in mammals and yeast, this happens immediately after scission events, the coat of plant CCVs appears to be retained and the components are only gradually discarded on their path to the TGN/EEs (Narasimhan et al., 2020).

In non-plant systems, the ATP−dependent dissociation of clathrin from CCV requires uncoating factors, which are the molecular chaperone heat shock cognate 70 (HSC70) and cofactor proteins like auxilin (Eisenberg and Greene, 2007). In plants, the auxilin-related protein-1 (AUXILIN-LIKE1) stimulates vesicle uncoating in the presence of HSC70 and interacts with SH3P1 and clathrin (Lam et al., 2001). AUXILIN-LIKE1 and 2 possible function in CME as they are part of a complex with clathrin, the ANTH domain protein CAP1, as well as SH3P2 and overexpression of both AUXILIN-LIKE1 and 2 leads to inhibition of endocytosis, most likely by preventing clathrin recruitment to endocytic pits. Nevertheless, they are not essential for endocytosis or development, implying that uncoating in plants may work differently (Adamowski et al., 2018).

### Adaptor Proteins in Plants

#### Multimeric Adaptors

Two types of multimeric adaptors exist in plant endocytosis, the evolutionarily conserved AP complexes and the plant (and some ameba)-specific TPLATE complex (Figure 2) (Zhang et al., 2015).

##### Adaptor protein complexes

There are five hetero-tetrameric AP complexes in *Arabidopsis thaliana*, which are involved in post-Golgi and endosomal vesicular trafficking and each (except for the *At*AP-5; Hirst et al., 2011) is composed of four subunits: two large, one medium, and one small (Chen et al., 2011; Hirst et al., 2011; Zhang et al., 2015). The AP-2 complex functions in CME (Bashline et al., 2013; Di Rubbo et al., 2013; Kim S. Y. et al., 2013), where it binds clathrin, cargo proteins and PtdIns(4,5)P_2_ at the PM (Honing et al., 2005; Fan et al., 2013). The PM association of CHC as well as the endocytic trafficking of the auxin efflux facilitators PIN1 (PIN-FORMED 1) and PIN2 is sensitive to PtdIns(4,5)P_2_ production, indicative of its involvement in CME (Ischebeck et al., 2013). Plants impaired in AP-2 subunits showed generally altered endocytosis, in particular of the PINs, brassinosteroid receptor BRASSINOSTEROID INSENSITIVE 1 (BRI1) and the borate efflux transporter BOR1 and exhibited pleiotrophic defects in plant growth and development (Di Rubbo et al., 2013; Fan et al., 2013; Kim S. Y. et al., 2013; Yoshinari et al., 2019). AP-2-dependent endocytosis maintains the polar localization of BOR1 under low-boron conditions, whereas the boron-induced vacuolar sorting of BOR1 is mediated through an AP-2-independent endocytic pathway (Yoshinari et al., 2019). Nevertheless, while the AP-2 complex is essential for early embryonic development in mammals (Mitsunari et al., 2005), *Arabidopsis* mutants of individual AP-2 subunits remain viable (Bashline et al., 2013; Fan et al., 2013; Kim S. Y. et al., 2013).

##### The TPLATE complex

Nucleation for CME may be different in plants, as they have retained an additional ancestral adaptor complex, the TPLATE complex. This essential component in the early CME events consists of eight core subunits (TPLATE, TASH3, LOLITA, TWD40-1, TWD40-2, TML, AtEH1, and AtEH2) (Gadeyne et al., 2014) and assembles at nucleation sites at the PM preceding AP-2 or at alternative unique sites to AP-2 and subunits of TPLATE complex recruit or stabilize AP-2 at the PM (Gadeyne et al., 2014; Wang et al., 2016). Thus, the TPLATE complex functions as an early multimeric adaptor complex in CME with overlapping, but also distinct, functions compared to the AP-2 complex (Zhang et al., 2015).

The TPLATE complex interacts with both the heavy and the light chain of clathrin, the AP-2, proteins containing the ANTH domain and DRPs and is indispensable for plant development (Van Damme et al., 2006; Gadeyne et al., 2014; Zhang et al., 2015). Several TPLATE subunits contain conserved domains like the SH3 domain in TASH3, the EPS15 homology (EH) domain in AtEH1 and AtEH2, and the μ-homology domain of the AP-2 complex in TML. These are domains, which are generally involved in membrane interactions, cargo recognition, and binding and recruitment of accessory proteins, which are also present in the mammalian Eps15, intersectin, and muniscins. Thus, the TPLATE complex shows functional similarities to the nucleation modules in mammalian system and could function as CME nucleation complex (Figure 2) (Gadeyne et al., 2014; Zhang et al., 2015). The presence of specific domains in the TPLATE complex members suggests a common ancestral origin with proteins in other eukaryotes involved in membrane trafficking or a nucleation/adaptor complex (Gadeyne et al., 2014; Hirst et al., 2014). The TML subunit for example could have evolved into the muniscin protein family (Hirst et al., 2014), where a prominent member is the mammalian FCHo protein, which acts as nucleation point for CME (Henne et al., 2010; Cocucci et al., 2012).

#### Monomeric Adaptor Proteins

Additionally, plants have several monomeric adaptor proteins, these also help link the cargo and membrane lipids to clathrin and multimeric adaptor complexes and thereby play a crucial role in the initiation of CCPs (Figure 2) (Paez Valencia et al., 2016). A large number of E/ANTH-domain proteins are found in the *Arabidopsis* genome (Zouhar and Sauer, 2014) and of the six plant proteins with an ENTH domain, two have been functionally characterized. EPSIN1, functions in AP-1-dependent post-Golgi trafficking events (Song et al., 2006) and EPSIN2/EPSINR2, binds PtdIn(3)P and a subunit of the AP-3 complex as well as the AP-2 complex, albeit to a lesser degree, and might localize in the endomembrane system from the TGN to the multi-vesicular bodies (MVBs) (Lee et al., 2007).

The ANTH domain family is larger in *Arabidopsis* and the monomeric adaptor AP180 was the first ANTH domain protein to be characterized. AP180, which gives the domain its name ANTH, interacts with a subunit of the plant AP-2 complex, promotes the assembly of clathrin in CME and regulates CCV size (Barth and Holstein, 2004). For another three ANTH proteins, ECA1, ECA2, and ECA4, it was suggested that they function as adaptors of CCV formation at the cell plate and the PM (Song et al., 2012). AP180 and ECA2, were shown to have a strong affinity to PtdIns(4,5)P_2_ and phosphatic acid and play an important role in CME (Kaneda et al., 2019). ECA4 plays a crucial role in recycling cargos from the TGN/EE to the PM (Nguyen et al., 2018) and, like the other ANTH domain protein CAP1, interacts with TML, one of the core components of the TPLATE complex (Gadeyne et al., 2014). CAP1 furthermore is found in a complex, which might function in CME together with clathrin, SH3P2 as well as auxilin-like proteins (Adamowski et al., 2018).

Further monomeric adaptors include the EH domain containing proteins, of which there are five in *A. thaliana* (Miliaras and Wendland, 2004). The *N*-terminal EH domain containing proteins AtEH1 and AtEH2, are part of the TPLATE complex and have recently been shown to interact with actin, like their yeast homologs, localizing to ER-PM contact sites, where they regulate the formation of autophagosomes (Wang P. et al., 2019). There are two *C*-terminal EHD proteins in *Arabidopsis*. Knockdown of AtEHD1 delayed and overexpression of AtEHD2 inhibited endocytosis, however, detailed mechanisms have not been characterized (Bar et al., 2008). A *Zea mays* homolog ZmEHD1 was shown to physically interact with an AP-2 subunit at the PM and in the mutant, endocytosis was drastically reduced and ZmPIN1 localization altered (Wang Y. et al., 2019).

### Endocytic Sorting Signals in Plants

#### Linear Motifs

In animals, the AP-2 recognizes, the di-leucine and the tyrosine (YXXΦ) based sorting motif, (where Y is tyrosine, X is any amino acid, and Φ is a bulky hydrophobic residue) (Traub and Bonifacino, 2013). Both motifs have been identified in plant PM proteins, but only the tyrosine-motifs have so far been linked to endocytosis (Geldner and Robatzek, 2008). The YXXΦ motif is important for the internalization of several PM proteins. For the PIN auxin efflux facilitators, mutation of the tyrosine motif resulted in a loss of binding of PIN1 to AP-2 subunits and thus reduction of its endocytosis (Sancho-Andres et al., 2016) and for PIN2 also a reduction in its internalization and the mutant construct failed to fully rescue the *pin2* phenotype (Kleine-Vehn et al., 2011). The mutation of the tyrosine motif in the tomato pathogen-related receptor-like protein *Lycopersicon esculentum* ETHYLENE-INDUCING XYLANASE RECEPTOR (LeEIX2) abolished internalization of the receptor in response to EIX application and thus its ability to induce the hypersensitive response (Ron and Avni, 2004). BOR1 contains three tyrosine motifs of in its cytoplasmic domain, which are essential to maintain the proper polar distribution of the boron exporter (Takano et al., 2010). Mutants lacking medium (μ)- or small (σ)-subunits of the AP-2 complex, showed altered polar localization and constitutive endocytosis of BOR1 under low-boron conditions. Nevertheless, association of the AP-2 complex with BOR1, was independent of YXXΦ sorting motifs, and the rapid internalization/vacuolar sorting of BOR1 was not affected in these mutants (Yoshinari et al., 2019).

The tyrosine kinase inhibitor Tyrphostin A23 (TyrA23) is a drug that inhibits the interaction between the tyrosine motif of the cargo and subunits of the AP-2 (Banbury et al., 2003) and is commonly used to interfere with endocytosis in plants (Dhonukshe et al., 2007). Care has to be taken though, as TyrA23, might also inhibit CME due to its protonophore activity, which leads to acidification of the cytoplasm. This undermines its use as a specific inhibitor of cargo recognition by the AP-2 adaptor complex (Dejonghe et al., 2016).

#### Phosphorylation

The posttranslational modification phosphorylation has been documented as a cue for endocytosis, where it functions in regulating signal recognition (Bonifacino and Traub, 2003). It has a central regulatory function in the endocytosis of PM proteins in plants. Phosphorylation receptor-like-kinase (RLK) LYSIN MOTIF-CONTAINING RLK5 (LYK5) by the CHITIN ELICITOR RECEPTOR KINASE1 (CERK1) is induced by the fungal polysaccharide chitin, and triggers its internalization (Erwig et al., 2017). A point mutation in a potential phosphorylation site of the pathogen-related receptor FLAGELLIN-SENSING 2 (FLS2), decreases the receptor internalization, thus indicating that phosphorylation of FLS2 is required for its endocytosis (Robatzek et al., 2006; Mbengue et al., 2016). Direct metal binding to a histidine-rich stretch of the PM localized IRON-REGULATED TRANSPORTER 1 (IRT1), triggers its phosphorylation and facilitates the subsequent recruitment of the IRT1 degradation factor (IDF) E3 ligase IDF1. Thus, phosphorylation seems to be a prior requirement for ubiquitination, and both are needed for efficient endosomal sorting of IRT1 (Dubeaux et al., 2018). For the boric acid importer NODULIN 26-LIKE INTRINSIC PROTEIN5;1 (NIP5;1), phosphorylation enhanced AP-2-dependent CME and mediates the strong polar localization (Wang H. J. et al., 2017). The phosphorylation states of the auxin efflux facilitators, the PINs, governs their polar distribution to apical and basal PM domains (Adamowski and Friml, 2015), whether the endocytosis of PINs is modulated directly by phosphorylation remain unclear though (Luschnig and Vert, 2014).

#### Ubiquitination

**FIGURE 3:**
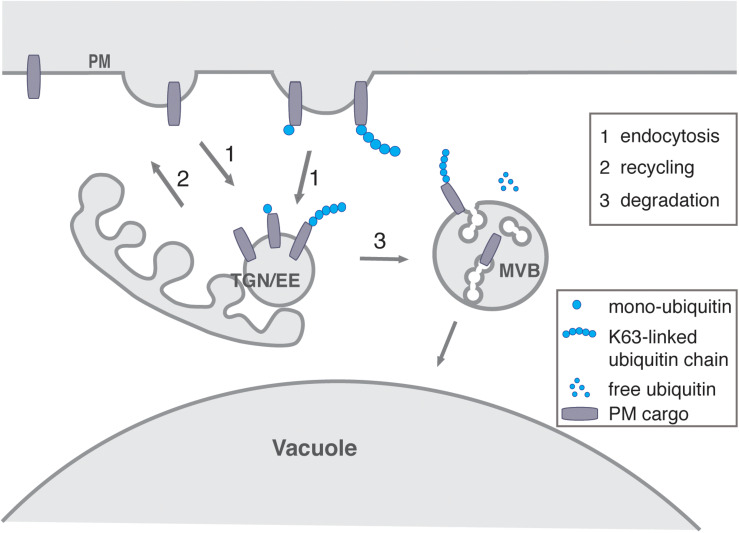
The role of ubiquitin in endocytosis and degradation. Continuous PM protein sorting from the PM to the TGN/EE occur regardless of its ubiquitination status (1) Alternatively, PM protein mono-ubiquination might promote endocytosis of the PM proteins to EE/TGN (1), whereas deubiquitination could favor recycling (2) back to the PM. K63-linked polyubiquitination of PM proteins might promote endocytic sorting from the PM to EE/TGN (1). Alternatively, the PM proteins could be subject to K63-linked polyubiquitination at endosomes and in any case polyubiquitination PM proteins would then be sorted via MVBs into the lytic vacuole. This step involves further ESCRT components (3).

The post-translation modification of PM proteins by ubiquitin serves as a signal for triggering their endocytosis and consequent sorting for degradation into intraluminal vesicles (ILVs) of MVBs. It thus plays a key role in directing the entry of PM proteins into the endosomal system (Figure 3) (Clague et al., 2012; Piper et al., 2014; Dubeaux and Vert, 2017). The enzymes and the mode of action of ubiquitination are conserved in eukaryotes and have been shown to play an important role in most aspects of plant development (Vierstra, 2012; Callis, 2014).

Ubiquitination is catalyzed by a series of consecutively acting enzymes: the ubiquitin activating enzyme (E1), the ubiquitin conjugating enzyme (E2), and the ubiquitin ligase (E3), which generate the formation of an isopeptide bond between the free amine of a lysine residue on the target protein and the *C*-terminus of ubiquitin (MacGurn et al., 2012). More than 1,500 ubiquitin-protein ligases are actively participating in this process, making ubiquitin conjugation an extraordinarily complex process in plants (Hua and Vierstra, 2011). Substrates can be mono-ubiquitinated, multiple mono-ubiquitinated, or decorated with ubiquitin chains. These chains result from linkage of one ubiquitin to the *N*-terminus or an internal lysine residue (K6, 11, 27, 29, 33, 48, and 63) of another ubiquitin moiety (Husnjak and Dikic, 2012; Romero-Barrios and Vert, 2018). Thus different types of chains, which can be linear or branched, can be formed, depending on which amino acid residues of the ubiquitin molecule are conjugated (Husnjak and Dikic, 2012). Two common forms are K48- and K63-linked chains, where the structure of the K48-linked di-ubiquitin is relatively compact, while K63-linked di-ubiquitin adopts an open conformation (Tenno et al., 2004; Varadan et al., 2004). K63-linked ubiquitin chains are the second-most abundant form of ubiquitination in plants (Kim D. Y. et al., 2013; Erpapazoglou et al., 2014; Tomanov et al., 2014; Romero-Barrios and Vert, 2018).

The E2/E3 pairing determines substrate specificity and type of chain linkage, although the same E3 may form different linkage types together with other E2s. Thus, the nature of the chain is defined by E2 enzymes (Tomanov et al., 2014; Stewart et al., 2016). *Arabidopsis* PM localized E3 RING DOMAIN LIGASES (RGLG), can form K63-linked chains together with the E2 UBC35, which appears essential for a wide range of developmental processes (Yin et al., 2007). Only recently, a clean knock out of the *Arabidopsis* E2s UBC35/36 unequivocally demonstrated their central roles as drivers of K63 polyubiquitin chain formation, underlining a key role for this type of protein modification (Romero-Barrios et al., 2020). Further studies on E2/E3 pairing, also revealed interactions between UBC35/36 and plant U-box (PUB) E3s (Turek et al., 2018). This large diversity in ubiquitination types is thought to allow specification of the respective roles in the different steps of down-regulation of membrane proteins, yet a unifying picture is still missing (Sigismund et al., 2004; d’Azzo et al., 2005; Lauwers et al., 2009; Komander and Rape, 2012; Dubeaux and Vert, 2017).

Ubiquitin binding domain (UBD)-containing proteins, or ubiquitin receptors, associate non-covalently with ubiquitin and translate the ubiquitin signals into a cellular response (Husnjak and Dikic, 2012). UBDs are typically short amino acid stretches, without a strict consensus sequence, that bind ubiquitin with a low binding affinity (Husnjak and Dikic, 2012). Several studies have added to deciphering the ubiquitome of plant (Saracco et al., 2009; Kim D. Y. et al., 2013; Svozil et al., 2014; Johnson and Vert, 2016; Walton et al., 2016; Aguilar-Hernandez et al., 2017; Romero-Barrios et al., 2020), enabling the cataloging of ubiquitin-modified proteins and building an excellent basis for future studies to understand this essential modification.

Ubiquitin chains can be altered or removed by deubiquitinating enzymes (DUBs) and in *Arabidopsis* there are around 50 putative DUBs that can be subdivided into five families (Isono and Nagel, 2014). Several E3 ligases involved in internalization processes occur in complexes with DUBs, which either remove or trim the ubiquitin signal contributing to its modulation. This enables tightly regulated and localized cycles of ubiquitination and deubiquitination to reverse or reinforce pathways, but also to prevent promiscuous or inappropriate events (Figure 3) (Hicke and Dunn, 2003; Urbe et al., 2012; Isono and Nagel, 2014).

Artificially ubiquitinated cargos, carrying in-frame fusions with ubiquitin showed enhanced internalization, providing direct evidence for ubiquitination acting as principal signal for PM protein endocytosis in plants. This was shown for the auxin efflux facilitator PIN2 (Leitner et al., 2012a), the PM H+-ATPase (PMA) (Herberth et al., 2012), a synthetic PM-localized reporter protein (TMD23-RFP) (Scheuring et al., 2012) and the BRI1 receptor kinase (Martins et al., 2015), where the sole presence of ubiquitin on cargo appeared as a sufficient signal for PM protein endocytosis and further sorting to the vacuolar lumen for degradation. Furthermore, immunoprecipitation experiments showed ubiquitination of a range of PM proteins including PIN2 (Abas et al., 2006; Leitner et al., 2012a), FLS2 (Goehre et al., 2008; Lu et al., 2011), the blue light receptor Phot1 (Roberts et al., 2011), IRT1 (Barberon et al., 2011), BOR1 (Kasai et al., 2011), and BRI1 (Martins et al., 2015). Additionally, several regulatory determinants influence endocytosis of ubiquitinated PM proteins, as shown by recent publications (Lu et al., 2011; Martins et al., 2015; Dubeaux et al., 2018; Retzer et al., 2019; Yoshinari et al., 2019).

The ubiquitination pattern for several PM proteins has been analyzed and dissected in depth and its effect on the endosomal trafficking of the PM protein evaluated. The atypical receptor kinase STRUBBELIG (SUB) is ubiquitinated and internalized by CME and transported via the TGN/EE and MVBs to the vacuole for degradation (Gao et al., 2019). FLS2 is K63-linked polyubiquitinated by the E3 ubiquitin ligases PUB12 and PUB13 after sensing of the flagellin peptide flg22 (Lu et al., 2011; Spallek et al., 2013). These E3 ligases also ubiquitinate LYK5 (Liao et al., 2017). FLS2 and CERK are also ubiquitinated and thus targeted for degradation by the bacterial effector AvrPto, thereby adding to the virulence by eliminating these RLK from the cell periphery (Goehre et al., 2008; Gimenez-Ibanez et al., 2009).

Mono-ubiquitination of several lysine residues in the cytosolic loop of IRT1 by the E3 ligase IDF1 (Shin et al., 2013) controls its internalization from the PM to the TGN/EE (Barberon et al., 2011) and a ubiquitination-defective IRT1 variant accumulates to the outer PM domain and is unable to reach the TGN/EE (Barberon et al., 2011). Abundance and localization of IRT1 is not regulated by iron availability (Barberon et al., 2011), but by non-iron metals, where an excess of those triggers the extension of multi mono-ubiquitins into K63-linked ubiquitin chains by the E3 ligase IDF1 and promotes vacuolar targeting of the transporter (Dubeaux et al., 2018; Cointry and Vert, 2019).

Under boron-limiting conditions, BOR1 is localized at the PM in a polar manner toward the stele, but in response to a boron excess, it is mono- or di-ubiquitinated in the *C*-terminal tail, endocytosed and transported to the vacuole for degradation to avoid the toxicity of boron accumulation (Kasai et al., 2011; Yoshinari and Takano, 2017). Substitution of a single lysine with alanine reduced ubiquitination under high boron concentrations but still allowed for endocytosis, although it completely blocked the degradation of BOR1. Thus, ubiquitination is necessary for BOR1 translocation to MVBs and degradation in the vacuole, but not required for the endocytosis of BOR1 (Kasai et al., 2011).

NITROGEN LIMITATION ADAPTION (NLA) is an E3 ligase that polyubiquitinates the high-affinity phosphate transporters (PHT1) (Park et al., 2014) and thus limits their levels at the PM under phosphate-sufficient conditions (Bayle et al., 2011). NLA also ubiquitinates the nitrate transporter NRT1.7 causing its down-regulation at the PM (Liu et al., 2017). BRI1 is post-translationally modified by ubiquitin or K63-linked ubiquitin chains, which promotes BRI1 internalization from the PM by CME (Di Rubbo et al., 2013) and is essential for its recognition at the TGN/EE for vacuolar targeting (Martins et al., 2015). Multi-monoubiquitination causes internalization of the photoreceptor Phot1 from the cell surface under low blue light, but does not control its degradation (Roberts et al., 2011).

For the auxin efflux facilitator PIN2, a constitutively ubiquitinated version was endocytosed while a lysine deficient allele, *pin2K-R*, was no longer ubiquitinated and failed to be degraded in the vacuole (Leitner et al., 2012a). The in-frame fusion of this lysin deficient allele with ubiquitin, was still constitutively endocytosed but no longer sorted into the vacuolar compartment, strongly suggesting a requirement of K63-linked ubiquitination for efficient vacuolar targeting of PIN2 while mono-ubiquitination signals its endocytosis from the PM (Leitner et al., 2012a, b). In a plant line lacking both RGLG1 and 2, PIN2 ubiquitination was decreased, making it likely that these E3 ligases control PIN ubiquitination. This PIN2 polyubiquitination is dependent on certain stimuli, thus determining stability of PIN2 by influencing the rate of its vacuolar targeting (Leitner et al., 2012a).

Another single-subunit RING-type E3 ubiquitin ligase, RSL1 K63-polyubiquinates the abscisic acid (ABA) receptors PYL4 and PYR1 at the PM, targeting them to the vacuolar degradation pathway (Bueso et al., 2014), this process involves CME and trafficking of the receptors via the endosomal sorting complex required for transport (ESCRT) pathway (Belda-Palazon et al., 2016; Yu F. et al., 2016; Garcia-Leon et al., 2019).

**FIGURE 4:**
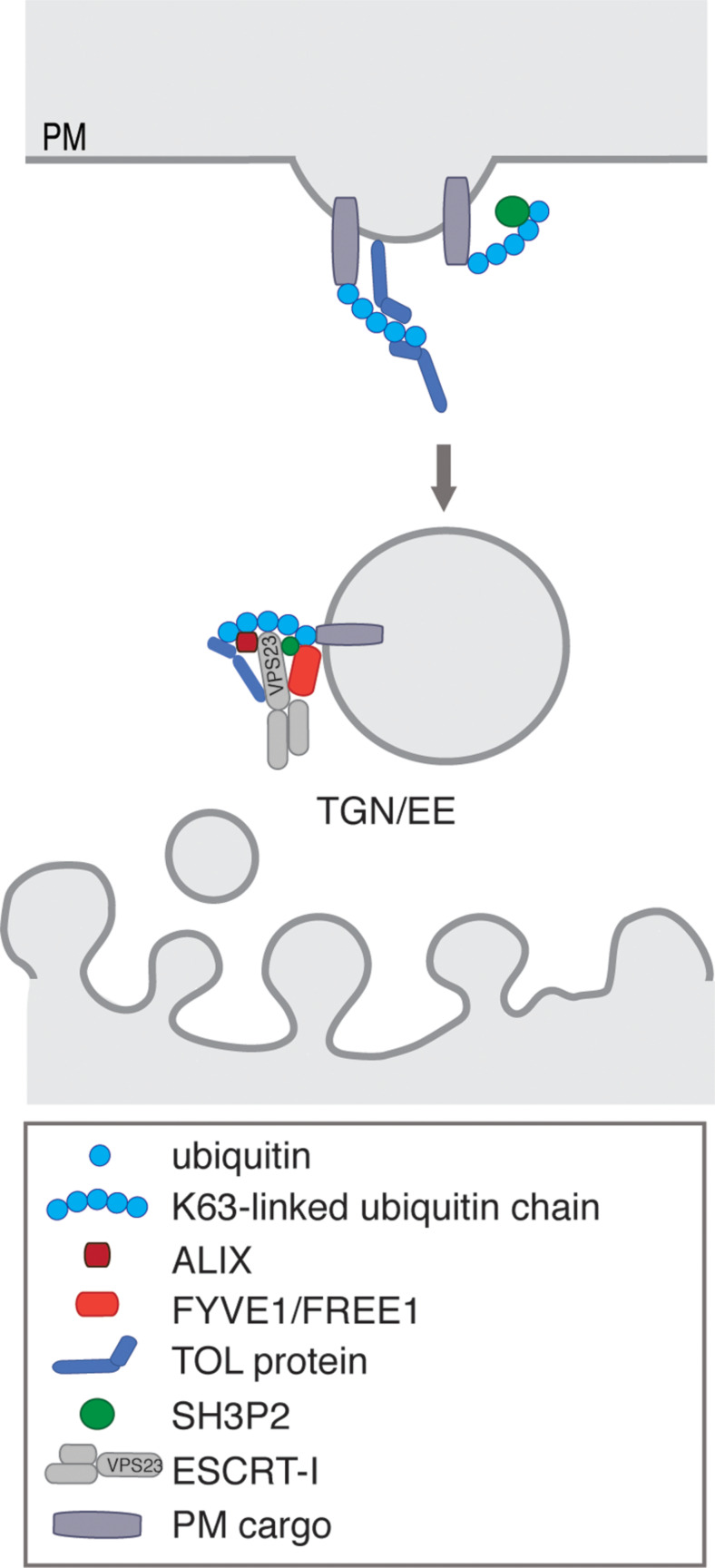
Close up of the endosomal trafficking from the PM toward TGN/EE. TOLs and SH3P2 fulfill ubiquitin recognizing functions at the PM, possibly guiding K63-linked ubiquitinated cargo to the endosomal membrane and interacting with/recruiting ESCRT-I subunits. At the endosomal membrane, the ESCRT-I binding FYVE1/FREE1 and ALIX also shows interaction with ubiquitin. This reveals the complex synergy of several possible ubiquitin receptors in the ESCRT-mediated degradation pathway.

It is evident that ubiquitination is involved in endocytosis and degradation of PM proteins in plants (Dubeaux and Vert, 2017), yet the direct mechanism of how ubiquitinated proteins are sorted into CCVs is still unclear. Interfering with CME by tampering with CHC function causes defect in the endocytosis of prominent PM proteins like the PINs, whose endocytosis is also controlled by ubiquitination (Luschnig and Vert, 2014), but the direct proof that disruption of CME causes accumulation of ubiquitinated cargos at the PM is still missing. In ubiquitin triggered CME, recruitment of ubiquitinated cargos into CCPs requires endocytic adaptor proteins, which contain UBDs. In mammals, monomeric endocytic adaptors proteins such as Eps15 and epsin, which additionally components of the CME machinery (Hawryluk et al., 2006), fulfill this function and collaborate with the ESCRT machinery to further sort cargos for degradation (Haglund and Dikic, 2012; Mayers et al., 2013; Schuh and Audhya, 2014). In contrast, the plant E/ANTH domain proteins, do not contain conserved UBDs, required for the interaction with ubiquitin (Holstein and Oliviusson, 2005; Zouhar and Sauer, 2014), indicating that ubiquitinated proteins are recognized at the PM by other plant-specific accessory adaptors recruiting cargos to the endocytic machinery (Figure 4). Endocytosis of the constitutively ubiquitinated PM cargos like PIN2 (Leitner et al., 2012a, b), PMA (Herberth et al., 2012), and BRI1 (Martins et al., 2015) depend on a hydrophobic patch centered around isoleucine 44 of ubiquitin, which is essential for the binding of ubiquitinated cargos by adaptor proteins containing UBDs (Dikic et al., 2009). For ubiquitin versions, where this isoleucine is mutated to an alanine (I44A), constitutive endocytosis of the ubiquitin-chimera is abolished (Leitner et al., 2012a; Korbei et al., 2013). Thus, internalization and degradation of constitutively ubiquitinated cargo involves their recognition proteins with UBDs. TARGET OF MYB1 (TOM1)-LIKE (TOL) proteins and SH3P2, are ubiquitin-binding protein that bind and transfer ubiquitinated proteins to the ESCRT machinery (Korbei et al., 2013; Nagel et al., 2017; Moulinier-Anzola et al., 2020) and might therefore compensate for the function in the endocytic adaptors that recognize ubiquitinated PM cargos and incorporate them into the nascent CCPs (Figures 2, 4).

### Other Plant Proteins in Clathrin-Mediated Endocytosis

Additional plant proteins play a role in CME, although their precise mechanisms are not known yet. STOMATAL CYTOKINESIS DEFECTIVE (SCD) 1 and 2, colocalize with CLC and co-fractionate with CCV (McMichael et al., 2013). The peripheral protein AT14A-LIKE 1 (AFL1), promotes growth during water stress and drought and localizes to the PM. It colocalizes with AP-2 and CLC and may function in endocytosis by regulating of actin filament organization (Kumar et al., 2015, 2019).

## Recycling

Once internalized from the cell surface, not all endocytosed membrane proteins are transported for degradation to the vacuole but escape degradation by recycling back to the PM (Figures 1, 3). (Paez Valencia et al., 2016). Recycling could thus enable a faster response to environmental changes without costly investments into *de novo* protein synthesis (Clague and Urbe, 2006). Plants do not possess endosomes specifically dedicated to recycling, but rather utilize the infrastructure provided by the TGN/EE and possibly early stages of the MVBs (Robinson and Neuhaus, 2016). The recycling machinery includes the small GTPases and their regulators as well as the retromer complex, while the ESCRT complex is responsible for transporting PM proteins to their degradation (Rodriguez-Furlan et al., 2019). The function of the retromer has been comprehensively reviewed very recently in (Rodriguez-Furlan et al., 2019).

### Small GTPases

Small GTPases are monomeric proteins that bind and hydrolyze GTP to GDP, which in turns requires Guanine nucleotide exchange factors (GEFs) that facilitate GDP-to-GTP exchange. Small GTPases represent master regulators of membrane trafficking, as they activate downstream effectors and are important contributors to organelle identity (Uemura and Ueda, 2014; Fan et al., 2015; Paez Valencia et al., 2016). The small GTPases of the ADP-ribosylation factor (ARF) family function in the recycling of internalized PM proteins. Plants contain eight ARF-GEFs, classified into two subfamilies: the GBF subfamily, which includes the fungal toxin Brefeldin A-sensitive GNOM, and the BIG subfamily (Geldner et al., 2003; Richter et al., 2007). GNOM, localizes to a not well characterized endosomal compartment and acts directly on recycling of cargo (Geldner et al., 2003; Richter et al., 2007). GNOM has also been shown to localize to the Golgi apparatus though and thus plays a role in trafficking from the ER to the Golgi apparatus, allowing to speculate about indirect roles in endosomal recycling, via affecting the integrity of the TGN (Naramoto et al., 2014).

### Deubiquitinating Enzymes and the ESCRT Machinery

A summary on the current knowledge an essential determinants for protein recycling is given in: Rodriguez-Furlan et al. (2019). Nevertheless, we would like discuss the effects of the ubiquitination status on protein fate, as proteins trafficking to the vacuole evade degradation by altering their ubiquitin modifications through DUBs (Isono and Nagel, 2014; Dubeaux and Vert, 2017).

Ubiquitination and deubiquitination occur constantly along the endosomal pathway and in mammals, several DUBs interact with ESCRT components to counterbalance the ubiquitination status of PM proteins destined for degradation. These ESCRT-DUBs interactions do not only control receptor fate through altering the ubiquitin code of the cargo at the initial steps, but also by influencing the function of the sorting complex (Clague and Urbe, 2006; Clague et al., 2019). Another important function of ESCRT associated DUBs is the deubiquitination of cargos that are already committed to inclusion into ILVs. Here, the DUBs degradation of alpha 4 (Doa4)/associated molecule with the SH3 domain of STAM (AMSH) in yeast/mammals function in the finals steps of the ESCRT pathway to deubiquitinate the cargo in order to maintain free ubiquitin levels (Clague and Urbe, 2006; Henne et al., 2011; Bissig and Gruenberg, 2014).

In the initial steps of the ESCRT pathway, the non-selective DUB ubiquitin-specific protease 8 (USP8/UBPY) and the stringent K63-linked chain selective DUB AMSH compete for binding to an ESCRT-0 subunit (Clague et al., 2019). The function of USP8 is to deubiquitinate and stabilize the ESCRT-0 complex (Row et al., 2006), while the K63-directed activity of AMSH promotes the deubiquitination and thus recycling of the cargo back to the PM (McCullough et al., 2004). Other reports also claim that AMSH may also directly regulate the function of the endocytic machinery, by altering the ubiquitination status and thus the activity of its components (Sierra et al., 2010). Other DUBs associated with the ESCRT-0 complex affect the ubiquitination status of the subunits, altering the ability of the ESCRT-0 subunits to bind to ubiquitin (Hoeller et al., 2006). USP8 also associates later on with ESCRT components, where it controls the ubiquitination state of one of the ESCRT-III subunits, charged multivesicular body protein (CHMP) 1B. This may promote its assembly into a membrane-associated polymer, which is needed for the budding of ILVs and thus represents a checkpoint for the temporal and spatial assembly of the ESCRT machinery (Crespo-Yanez et al., 2018). DUBs therefore interact with ESCRT components to modulate the ubiquitination status of cargos, or to that of the components of the machinery to control the function of the ESCRT complex and the fate of the ubiquitinated cargo (Wright et al., 2011).

*Arabidopsis* contains three AMSH-like proteins, where AMSH 1 and 3 associate with subunits of the ESCRT machinery and show a specificity toward K63-linked ubiquitin chains, but can also trim K48 linked chains (Isono et al., 2010). AMSH1 interacts with the ESCRT-III-subunit VPS2.1 are plays a role in autophagic degradation, its knockdown mutant does not have an apparent growth defect (Katsiarimpa et al., 2013). AMSH3 on the other hand is essential for seedling development and proper vacuolar trafficking of both biosynthetic and endocytosed cargo. It also interacts with ESCRT-III subunits VPS2.1 and also VPS24.1. AMSH3 and another ESCRT component, SUPPRESSOR OF K^+^ TRANSPORT GROWTH DEFECT 1 (SKD1) compete for binding to VPS2.1 and this interaction is important for deubiquitination of ubiquitinated membrane substrates prior to their degradation in plants (Katsiarimpa et al., 2011, 2014). AMSH3 is recruited to endosomes through a direct interaction with apoptosis-linked gene-2 interacting protein X (ALIX), a conserved ESCRT-related protein that binds membranes, ubiquitin, and ESCRT components (Kalinowska et al., 2015). Furthermore, AMSH3 associates with SH3P2 and an ESCRT-I subunit on CCV (Nagel et al., 2017). Although AMSH proteins display DUB activity *in vitro*, and ubiquitin-binding proteins like SH3P2 could facilitate the access of DUBs to ubiquitin chains early on in the endocytic pathway, no plant DUB has so far been directly associated with deubiquitination of cargo for recycling (Isono and Nagel, 2014).

## Degradative Sorting

Proteins that are not recycled back to the PM from the TGN/EE are destined for degradation and proceed further downstream in the endocytic pathway to the vacuole (Paez Valencia et al., 2016; Otegui, 2018). The ESCRT machinery is an evolutionarily conserved, multi-subunit membrane remodeling complex, with the ability to form membrane budding away from the cytosol. It is therefore very different from the machineries involved in the curvature into the cytosol as in the formation of the CCVs and most other vesiculation events (Paez Valencia et al., 2016; Gao et al., 2017; Isono and Kalinowska, 2017). As the endosomal membranes of the TGN/EEs become capable of associating with ESCRT proteins, they start internalizing portions of their limiting membrane into ILVs, and then gradually mature into MVBs, that ultimately fuses with vacuoles, releasing their contents for degradation (Otegui, 2018).

### The ESCRT Machinery

The ESCRT machinery plays an essential role in the biogenesis of the MVBs and the sorting of ubiquitinated membrane proteins for degradation (Henne et al., 2011; Paez Valencia et al., 2016; Gao et al., 2017; Isono and Kalinowska, 2017). This starts with cargo recognition and recruitment of the remodeling machinery in a stepwise process, performed by a series of protein complexes termed ESCRT-0 to ESCRT-III and various accessory components, which act in recognition, concentration and sequestering of ubiquitinated cargo into the ILVs and the membrane deforming events essential for this procedure (Henne et al., 2011; Paez Valencia et al., 2016; Gao et al., 2017; Isono and Kalinowska, 2017). The ESCRT-0 complex is required for initial targeting and concentration of ubiquitinated cargo and further recruits the ESCRT-I, to which it passes on the ubiquitinated cargo (Hurley, 2010). The ESCRT-I further recruits the ESCRT-II to endosomes and the presence of both complexes induces the invagination of the limiting membrane toward the endosomal lumen (Hurley, 2010). ESCRT-I and II complexes could also act in parallel to concentrate the ubiquitinated cargo (Hurley, 2008; Hurley and Ren, 2009). The upstream components recruit, activate, and organize the polymerization of ESCRT-III, which does not contain any known UBD. The ubiquitin molecule is removed from the cargo proteins by DUB activity, once the ESCRTs are assembled. In a final step, an AAA ATPase, whose recruitment and activity is mediated by ESCRT-III related proteins, recycles the ESCRT-III back into its monomeric form (Hurley and Hanson, 2010; Schoneberg et al., 2017).

#### ESCRT-0

The ESCRT-0 complex, which binds and recruits the ESCRT-I, is the made up of two subunits the Vps27/hepatocyte growth factor-regulated tyrosine kinase substrate (HRS) and the Hse1 (Hbp STAM, EAST 1)/signal transducing adaptor molecule (STAM) in yeast/humans (Raiborg and Stenmark, 2009; Henne et al., 2011). It is found at EE and at the PM, although the precise starting point for the interaction of ESCRT-0 with ubiquitin is still under debate (Mayers et al., 2013). It is suggested to preassemble with cargos at the PM at CCPs, where it enhances the efficiency of sorting-events, without affecting the CCVs formation (Mayers et al., 2013). The core structure of the ESCRT-0 complex is made up of two antiparallel intertwined GAT (GGA and TOM1) domains flanked by further α-helical UBDs. This higher order multimeric structure, with its conformational plasticity, allows for recognition of different ubiquitinated cargos and provides a strong ubiquitin-binding platform (Ren and Hurley, 2010; Piper et al., 2014). The ESCRT-0 associates preferentially with K63-linked ubiquitin chains and blocks their binding to proteasomes, thus preventing proteasomal degradation and enhancing lysosomal/vacuolar degradation of cargo proteins (Nathan et al., 2013). Some ESCRT-0 subunits can recruit DUBs, allowing for modulation in the ubiquitination pattern of the cargo as well as of subunits of the machinery (Raiborg and Stenmark, 2009; Wright et al., 2011). The ESCRT-0 interacts with clathrin and this binding to the flat clathrin lattice restricts ESCRT-0 distribution on the endosomes and allows for the clustering of ubiquitinated cargo into microdomains (Schuh and Audhya, 2014). Furthermore, targeting to EEs, is assisted by the Fab 1, YOTB, Vac 1, and EEA1 (FYVE) domains of Vps27/HRS, which, in yeast/mammals binds the EE associated PtdIn(3)P (Hurley, 2010).

While the other ESCRT machinery complexes are ubiquitous in eukaryotes and ancient in origin, the ESCRT-0 appears to be a more recent addition and is as such not present in plants (Winter and Hauser, 2006; Leung et al., 2008). Potential candidates to substitute for this complex involve the TOM1 protein family, which share the same tandem array of *N*-terminal domains, the VHS (Vps27/HRS/Stam) and GAT domain, which are able to bind ubiquitin and potentially membranes (Mosesso et al., 2019). A complex centering around TOM1, functioning either parallel, as in mammals (Wang et al., 2010) or alternatively, as in ameba (Blanc et al., 2009), to the ESCRT-0 complex, has been described. TOM1L1 has also been proposed to package ubiquitinated PM proteins into CCVs (Liu et al., 2009). TOM1 is widely conserved in eukaryotes and from its phylogenetic distribution, it is likely to have been either replaced or perhaps supplemented by ESCRT-0 in opisthokonts (Herman et al., 2011). Additionally, other plant candidates, with similar functional attributes to the ESCRT-0 could also substitute the elusive ESCRT-0 complex in plants (Mosesso et al., 2019).

#### ESCRT-I

The ESCRT-I complex forms an elongated hetero-tetrameric complex in yeast/mammals, which contains one copy each subunit: Vps23/tumor susceptibility gene-101 (TSG101), VPS28, VPS37, and MVB sorting factor of 12 kDa (MVB12) or the Mvb12-like subunit ubiquitin-associated protein 1 (UBAP1) (Schuh and Audhya, 2014). The Vps23/TSG101 subunit has a UBD at its *N*-terminus, the ubiquitin E2 variant (UEV) domain. This domain has a dual function as it is responsible the binding the ubiquitinated cargo and a subunit of the ESCRT-0, via the P[S/T]AP motif in ESCRT-0 (Bache et al., 2003; Hicke and Dunn, 2003). Furthermore, the VPS28 is responsible for binding of the ESCRT-II via its *C*-terminus (Kostelansky et al., 2007).

*Arabidopsis* contains two isoforms of each of the ESCRT-I subunits VPS23 (ELC/VPS23A and VPS23B), VPS28 (VPS28-1 and VPS28-2), and VPS37 (VPS37-1 and VPS37-2), but no obvious Mvb12-like proteins (Winter and Hauser, 2006; Leung et al., 2008). VPS23A binds ubiquitin and associates with VPS37 and VPS28 in a putatively intact plant ESCRT-I complex. It participates in trichome development and cell division (Spitzer et al., 2006) and was found to be important in ABA signaling by affecting the subcellular localization and stability of ABA receptors via vacuole-mediated degradation (Yu F. et al., 2016). *vps28-2* and *vps37-1* mutant plants display altered endosomal sorting of the pathogen-related receptor FLS2 and are compromised in pathogen responses, but develop normally otherwise, suggesting functional redundancy with the other VPS28 and VPS37 isoforms (Spallek et al., 2013).

#### ESCRT-II

The ESCRT-II links the upstream ubiquitin-binding ESCRT complexes to the downstream ESCRT-III complex and is made up of the subunits VPS22, VPS25, and VPS36, which assemble in a Y-shaped 1:2:1 hetero-tetramer (Hurley, 2010). All ESCRT-II subunits are present in single copies in *A. thaliana* (Winter and Hauser, 2006; Leung et al., 2008; Richardson et al., 2011). The *vps22* mutant in rice causes seedling lethality, yet whether endosomal sorting is affected remains to be determined (Zhang et al., 2013). VPS36 binds ubiquitin and forms a putative ESCRT-II with VPS22 and VPS25. It is essential for MVB formation, vacuolar biogenesis and the endosomal sorting of several PM proteins into the vacuole for degradation (Wang S. L. et al., 2017).

#### ESCRT-III

The ESCRT-III, which does not bind ubiquitinated cargos anymore, is critical for membrane scission during sorting of the cargo into ILVs (Henne et al., 2012). For plant ILVs it was recently shown that they form networks of concatenated vesicles that bud in the lumen of MVBs instead individual vesicles. These concatenated ILVs remain connected by narrow bridges, thus trapping the cargo and the ESCRT-III could act as diffusion barriers to prevent the escape of the cargos destined for degradation (Buono et al., 2017).

Membrane recruitment of the ESCRT-III is initiated by binding to ESCRT-II subunit VPS25 to VPS20, when inactive monomers from the cytosol polymerize into the active ESCRT-III (Hurley, 2010; Schuh and Audhya, 2014). In yeast/animals the core ESCRT-III consists of four small, highly charged subunits, which polymerize into long spiral filaments on highly curved membranes: Vps2p/CHMP2, Vps20p/CHMP6, Vps24p/CHMP3, and Snf7p (Sucrose Non-Fermenting 7)/CHMP4, (Shen et al., 2014). Furthermore, there are three accessory proteins, Did2 (Doa4-independent degradation 2)/CHMP1, Vps60/CHMP5, and increased salt tolerance 1 (IST1). IST1 modulate the activity of the ESCRT-III complex, as well as its association with the Vps4/SKD1 complex (Raiborg and Stenmark, 2009).

The ESCRT-III machinery is conserved in plant cells (Cai et al., 2014) and all core subunits in *Arabidopsis*, have two homologs, except for VPS2, which has three (Winter and Hauser, 2006), however, only VPS2.1 functions as a classical ESCRT subunit and binds the DUB AMSH3 (Katsiarimpa et al., 2011). Transient over-expression of dominant negative core ESCRT-III subunits resulted in defects in vacuolar biogenesis and MVB formation with less ILVs (Cai et al., 2014).

The accessory ESCRT-III related *Arabidopsis* protein CHMP1 interacts with the Vps4/SKD1 complex to regulate MVB biogenesis. The *chmp1a/chmp1b* double-mutants of the two functionally redundant *CHMP1* genes, exhibits embryonic or early seedling lethality and fail to sort PIN1 into ILVs (Spitzer et al., 2009). The maize ortholog supernumerary aleurone layer 1 (SAL1), regulates endosomal sorting and degradation of PM proteins and is therefore necessary for aleurone endosperm differentiation (Shen et al., 2003; Tian et al., 2007). For the *Arabidopsis* homolog of yeast IST1, there are 12 IST1-like proteins (ISTL1–12), however, only ISTL1 has been shown to function in the ESCRT pathway, where it interacts with Lyst-interacting protein 5 (LIP5), SKD1 and CHMP1A and is predicted to regulate MVB biogenesis and sorting of membrane proteins for degradation (Buono et al., 2016).

#### Vps4/SKD1 and Accessory Proteins

Through ATP hydrolysis, the AAA ATPase Vps4/SKD1, disassembles the ESCRT-III complex, allowing for recycling of the ESCRT III subunits. This step is crucial for the completion of the membrane scission in the formation of ILVs, after which MVBs can fuse with the vacuole/lysosomes, where ILVs are released for degradation (Hurley, 2010; Schuh and Audhya, 2014). Vps4/SKD1 interacts with VPS 20-associated 1 (Vta1)/LIP5, which stabilizes its oligomeric conformation, thus functioning as a positive regulator for its activity (Schuh and Audhya, 2014).

In *Arabidopsis*, there is only one Vps4/SKD1 homolog named SKD1, which localizes to the cytoplasm and MVBs. Its ATPase activity is positively regulated by LIP5 (Haas et al., 2007), and disruption of *SKD1* is lethal, while *lip5* mutants are normal in growth and development. Though LIP5 is not necessary for plant survival, it is essential for normal responses to biotic and abiotic stresses (Haas et al., 2007; Wang et al., 2014, 2015). A SKD1 ortholog from *Zea mays* (ZmSKD1), which is up-regulated by salt or drought stress, interacts with NtLIP5 (LIP5 from *Nicotiana tabacum*) (Xia et al., 2013). PROS (POSITIVE REGULATOR OF SKD1) is a flowering plant-specific ESCRT protein that interacts with SKD1 and increases its ATPase activity *in vitro*. It is thus, like LIP5, another positive regulator of the SKD1, although the two are structurally different. Silencing of PROS leads to reduced cell expansion and abnormal organ growth (Reyes et al., 2014).

Another ESCRT related protein is the yeast/mammalian bypass of C kinase 1 (BCK1)-like resistance to osmotic shock 1p (Bro1)/ALIX. Both were shown to interact with ESCRT-III, however, only the mammalian ALIX, also interacts with ESCRT-I subunits, therefore having the potential to bridge the ESCRT-I and ESCRT-III complexes (Bissig and Gruenberg, 2014). Loss of Bro1 affects ILV formation, as it regulates the membrane-scission activity of ESCRT-III via binding to Snf7 (Wemmer et al., 2011). Bro1/ALIX bind ubiquitin, specifically K63-linked ubiquitin (Dowlatshahi et al., 2012), suggesting they may function as ubiquitin receptor for protein sorting into MVBs, in addition to ESCRT-0 (Pashkova et al., 2013; Mosesso et al., 2019).

In *Arabidopsis* the conserved ESCRT-related protein ALIX (Figure 4), binds membranes, ubiquitin, the ESCRT-I subunit VPS23A and the ESCRT-III subunit SNF7 and is indispensable for the biogenesis of the vacuole and MVB and thus for plant growth and development (Cardona-Lopez et al., 2015; Kalinowska et al., 2015; Shen et al., 2018). ALIX recruits AMSH3 to endosomes through direct interaction (Kalinowska et al., 2015). Furthermore, ALIX, mediates trafficking to the vacuole of PHT1, BRI1 but also ABA receptors, to which ALIX can even bind directly (Cardona-Lopez et al., 2015; Garcia-Leon et al., 2019). ALIX associated with VPS23A and FYVE1/FREE1 to be incorporated into the ESCRT-I complex and thus may also function in bridging ESCRT-I and ESCRT-III complexes in plants (Shen et al., 2016).

### Plant-Specific ESCRT Components

In plants, there are several plant unique components with limited protein sequence similarity to the mammalian/yeast ESCRT subunits that interact with known plant ESCRT subunits and help shape and regulate ESCRT-dependent processes.

#### SH3 Domain Containing Protein-2

The ubiquitin binding protein SH3P2 belongs, together with SH3P1 and SH3P3, to a family of three proteins in *A. thaliana*, with a central BAR domain, acting in membrane curvature generation and present in proteins involved in CME and a *C*-terminal SH3 domain, which functions in protein–protein interactions (Lam et al., 2001; Zhuang et al., 2013). It has been implicated in as vacuolar trafficking of ubiquitinated cargos and has been shown to bind K63-linked ubiquitin, the ESCRT-I subunit VPS23A and the DUB AMSH3. It further localizes to CCV and could thus also function as an ESCRT-0 substitute (Figure 4) (Kolb et al., 2015; Nagel et al., 2017; Mosesso et al., 2019). SH3P2 was further shown to interact with CLC1 and found in a complex potentially involved in CME with two putative homologs of the CCV uncoating factor auxilin and the ANTH domain protein CAP1 (Adamowski et al., 2018).

#### FYVE1/FREE1

An ESCRT-I-related component called FYVE1/FREE1 (FYVE DOMAIN PROTEIN REQUIRED FOR ENDOSOMAL SORTING 1) (Figure 4) was identified in *Arabidopsis*, where it localizes to MVBs, binds ubiquitin, PtdIn(3)P, interacts with SH3P2, VPS23A, and VPS23B via its PTAP-like motifs and with SNF7 to be incorporated into the ESCRT-III complex, yet not with the TOLs. It is essential for the formation of ILVs, vacuolar biogenesis, autophagic degradation, seedling development, polar localization of the iron transporter IRT1 and degradation of PM proteins and ABA receptors (Barberon et al., 2014; Gao et al., 2014; Kolb et al., 2015; Belda-Palazon et al., 2016). Additionally, FYVE1/FREE1 has a non-endosomal function in attenuating ABA signaling, where following ABA treatment FYVE1/FREE1 is phosphorylate causing its nuclear import, where it interacts with the ABA-responsive transcription to reduce their binding to the *cis*-regulatory sequences of downstream genes (Li et al., 2019). FYVE1/FREE1 recruitment to the MVBs is regulated by a plant-specific Bro1-domain protein BRAF, which competes with FYVE1/FREE1 in its binding to VPS23A. Thus, BRAF functions as an ESCRT regulator, as its depletion increases FYVE1/FREE1 association with MVB membranes (Shen et al., 2018).

#### TOM1-Like Proteins

Many eukaryotic groups, do not have ESCRT-0 components and therefore need to rely on other proteins to initially recognize ubiquitinated cargo at the PM (Mosesso et al., 2019). *Arabidopsis* has a family of nine proteins, TOL1-9, with a domain organization similar to the ESCRT-0, demonstrated to be crucial in vacuolar targeting of ubiquitinated PM proteins (Korbei et al., 2013; Yoshinari et al., 2018; Moulinier-Anzola et al., 2020). Members of the TOL protein family, interact with ESCRT-I subunits and bind ubiquitin, with a pronounced substrate preference to K63-linked ubiquitin. This could be caused by the tandemly arranged UBDs in the *N*-terminus of all nine TOL proteins, as mutations of these domains resulted in complete loss of ubiquitin binding. These UBDs, could thus regulate fidelity and kinetics of the sorting of endocytosed ubiquitinated cargo and the TOLs could function as multivalent ubiquitin-binding complexes (Figure 4) (Moulinier-Anzola et al., 2020).

Interaction between TOLs and ubiquitinated PM proteins destined for degradation are not clear at present as TOLs were shown to exhibit differences in their subcellular localization, from PM- and EE/TGN-localized TOLs to cytoplasmic ones, indicating they might have activities in different subcellular sites (Moulinier-Anzola et al., 2020). TOL5, with its cytoplasmic localization has been described to function at MVBs, where it co-localizes with BOR1 on its route to the vacuole under high-boron conditions (Yoshinari et al., 2018), while TOL6 functions at the PM in the degradation of PIN2 (Korbei et al., 2013). As only higher order TOL knockouts sufficiently inhibit the down-regulation of PM-localized ubiquitinated proteins (Korbei et al., 2013; Moulinier-Anzola et al., 2020), the TOLs could function in a network in the plant endomembrane system, where they pass on the ubiquitinated cargo, but this of course awaits further assessment (Figure 4). TOL ubiquitin receptors are themselves ubiquitinated, which serves as a regulatory signal, influencing their sub-cellular distribution and thus could help to regulate the efficiency of the degradation of PM localized cargo (Moulinier-Anzola et al., 2020).

Plants have to be able to cope sensitively and accurately to their often times harsh environment and to adapt quickly to immanent changes. The necessity of the sessile plants to fine-tune and control the abundance of their PM proteins to be able to respond quickly and precisely is reflected in high number of plant-specific factors, next to the conserved canonical trafficking machinery, in the ubiquitin-dependent degradation of membrane proteins. Thus, many plant-specific proteins have been shown to be involved in the regulation of endosomal trafficking and endocytosis and some ESCRT subunits have undergone drastic gene expansions. Furthermore, recent publications point to extensive crosstalk of diverse signaling- and trafficking pathways, where specific ESCRT proteins may even fulfill additional roles (Gao et al., 2017; Cui et al., 2018; Vietri et al., 2020). Thus, to thoroughly understand the molecular mechanisms underlying the regulation of ESCRT-dependent degradation of ubiquitinated proteins will be of decisive importance to fully understand plant adaptation processes to a changing environment.

## Author Contributions

BK wrote the first draft of the manuscript. MS wrote sections of the manuscript. Both authors contributed to manuscript revision, read, and approved the submitted version.

## Conflict of Interest

The authors declare that the research was conducted in the absence of any commercial or financial relationships that could be construed as a potential conflict of interest.
